# O.3.2-1 Evaluation of the Active Together multi-modal cancer rehabilitation service embedded within clinical care

**DOI:** 10.1093/eurpub/ckad133.146

**Published:** 2023-09-11

**Authors:** Michael John Thelwell, Anna Myers, Liam Humphreys, Gabriella Frith, Katie Pickering, Gail Phillips, Carol Keen, Rob Copeland

**Affiliations:** Sheffield Hallam University, UK; Sheffield Hallam University, UK; Sheffield Hallam University, UK; Sheffield Hallam University, UK; Sheffield Hallam University, UK; Sheffield Hallam University, UK; Sheffield Teaching Hospitals NHS Foundation Trust; hwblh1@exchange.shu.ac.uk; Sheffield Hallam University, UK

## Abstract

**Purpose:**

The Active Together service provides multimodal rehabilitation support for people affected by cancer, embedded within clinical pathways at all stages of cancer treatment. The service aims to improve patient outcomes, such as physical fitness prior to surgery, tolerance to treatments, post-operative complications, and survival rates.

**Project description:**

The design of the Active Together service was informed by extensive stakeholder engagement, involving consultations with patients and health professionals to determine the key elements of an impactful and sustainable rehabilitation service. Informal discussion groups and co-design workshops were held to understand ‘what mattered most’ to patients and to develop key features of the service.

The Active Together service is delivered by a multi-disciplinary team at the Advanced Wellbeing Research Centre at Sheffield Hallam University, in partnership with Sheffield Teaching Hospitals and Yorkshire Cancer Research. Patients with a cancer diagnosis, over the age of 18 years and receiving treatment with curative intent are referred to the service, which provides exercise, nutritional and psychological support throughout their treatment.

The evaluation aims to determine the impact of the service on patient outcomes and any economic benefits to the wider healthcare system. Outcome measures are captured throughout the patients’ treatment to assess the impact on their physical, nutritional, and psychological state. Economic evaluation will assess its effect on the costs of patients’ treatment, compared to patients that received treatment for cancer without rehabilitation.

Initially, the Active Together service has provided support to patients in Sheffield, being treated for one of three specific forms of cancer: lung, colorectal and upper gastrointestinal. Over the next 2-3 years, the service will be scaled-up to provide support to patients receiving treatment for other forms of cancer at hospitals across Yorkshire. Findings will be disseminated to key stakeholders within the healthcare system and in academic journals.

**Conclusions:**

Evidence to support the role of multi-modal rehabilitation for cancer patients is growing. The translation of that evidence into practice is less advanced. The results of this evaluation will provide valuable insight into service implementation and an understanding of the impact of cancer rehabilitation on both patient outcomes and the health economic landscape.

